# Effects of dietary supplementation with dandelion tannins or soybean isoflavones on growth performance, antioxidant function, intestinal morphology, and microbiota composition in Wenchang chickens

**DOI:** 10.3389/fvets.2022.1073659

**Published:** 2023-01-04

**Authors:** Xiang Li, Ruiping Sun, Quanwei Liu, Yuanfang Gong, Yangkun Ou, Qi Qi, Yali Xie, Xiuping Wang, Chenjun Hu, Shouqun Jiang, Guiping Zhao, Limin Wei

**Affiliations:** ^1^Hainan Key Laboratory of Tropical Animal Breeding and Epidemic Research, Institute of Animal Husbandry & Veterinary Research, Hainan Academy of Agricultural Sciences, Haikou, China; ^2^Hebei Key Laboratory of Specialty Animal Germplasm Resources Exploration and Innovation, College of Animal Science and Technology, Hebei Normal University of Science & Technology, Qinhuangdao, China; ^3^Sanya Institute, Hainan Academy of Agricultural Sciences (Hainan Experimental Animal Research Center), Sanya, China; ^4^Hainan (Tanniu) Wenchang Chicken Co., Ltd., Haikou, China; ^5^Tropical Crop Genetic Resource Research Institute, Chinese Academy of Tropical Agricultural Sciences, Haikou, China; ^6^State Key Laboratory of Livestock and Poultry Breeding, Key Laboratory of Animal Nutrition and Feed Science in South China, Ministry of Agriculture and Rural Affairs, Guangdong Provincial Key Laboratory of Animal Breeding and Nutrition, Institute of Animal Science, Guangdong Academy of Agricultural Sciences, Guangzhou, China

**Keywords:** growth performance, biochemical blood indexes, antioxidant function, intestinal health, dandelion tannin, soybean isoflavones, Wenchang chicken

## Abstract

Many benefits have been found in supplementing tannins or soybean isoflavones to poultry, including increased body weight gain, antioxidant activity, and better intestinal morphology. However, few studies tested the influence of dandelion tannins or soybean isoflavones supplementation on Wenchang chickens. This study investigates the effects of dietary supplementation with dandelion tannins or soybean isoflavones on the growth performance, antioxidant function, and intestinal health of female Wenchang chickens. A total of 300 chickens were randomly divided into five groups, with six replicates per group and 10 broilers per replicate. The chickens in the control group (Con) were fed a basal diet; the four experimental groups were fed a basal diet with different supplements: 300 mg/kg of dandelion tannin (DT1), 500 mg/kg of dandelion tannin (DT2), 300 mg/kg of soybean isoflavone (SI1), or 500 mg/kg of soybean isoflavone (SI2). The experiment lasted 40 days. The results showed that the final body weight (BW) and average daily gain (ADG) were higher in the DT2 and SI1 groups than in the Con group (*P* < 0.05). In addition, dietary supplementation with dandelion tannin or soybean isoflavone increased the level of serum albumin (*P* <0.05); the concentrations of serum aspartate aminotransferase and glucose were significantly higher in the SI1 group (*P* < 0.05) than in the Con group and the concentration of triglycerides in the DT1 group (*P* < 0.05). The serum catalase (CAT) level was higher in the DT1 and SI1 groups than in the Con group (*P* < 0.05). The ileum pH value was lower in the DT2 or SI1 group than in the Con group (*P* < 0.05). The jejunum villus height and mucosal muscularis thickness were increased in the DT2 and SI1 groups (*P* < 0.05), whereas the jejunum crypt depth was decreased in the DT1 or DT2 group compared to the Con group (*P* < 0.05). In addition, the messenger RNA (mRNA) expression level of zonula occludens 1 (*ZO-1*) in the duodenum of the SI1 group and those of *occludin, ZO-1*, and *claudin-1* in the ileum of the DT2 and SI1 groups were upregulated (*P* < 0.05) compared to the Con group. Moreover, the DT2 and SI1 groups exhibited reduced intestinal microbiota diversity relative to the Con group, as evidenced by decreased Simpson and Shannon indexes. Compared to the Con group, the relative abundance of Proteobacteria was lower and that of *Barnesiella* was higher in the DT2 group (*P* < 0.05). Overall, dietary supplementation with 500 mg/kg of dandelion tannin or 300 mg/kg of soybean isoflavone improved the growth performance, serum biochemical indexes, antioxidant function, and intestinal morphology and modulated the cecal microbiota composition of Wenchang chickens.

## 1. Introduction

Wenchang chicken is a Chinese local breed with delicious meat and unique flavor (1). Antibiotics are used as feed additives, mixed with chicken feed, and given to this breed of Wenchang chicken to ward off diseases and to promote their growth (2). However, the abuse of antibiotics by way of overdose administered to chickens has led to the emergence of an entirely new crop of antibiotic-resistant bacteria that threaten and endanger human health (3). China has banned the use of adding antibiotics to chicken feed for the very same reason. However, noninclusion of antibiotics in the feed brings with it numerous issues that need to be sorted out to boost chicken production. First, the noninclusion of antibiotics in the feed renders the chickens vulnerable to bacterial attack; second, the prevention of disease in chickens is rendered inefficient; and third, the noninclusion of antibiotics in feed makes it expensive as the chickens become weak and fall sick. These ailing chickens have to be nursed back to health, which is costly, compared to the inclusion of antibiotics in feed which does not entail these extra costs and so is less expensive (4, 5). Therefore, it is extremely urgent to find alternative compounds to replace antibiotics in chicken production.

Plant extracts are natural bioactive compounds with green, safe, and efficient characteristics, and are widely used in animal production. It was reported that plant extracts can improve growth performance and intestinal histology and regulate the immune system (6, 7). There are many kinds of plant extracts, including flavonoids, essential oils, alkaloids, polyphenols, and polysaccharides (8). Specifically, soybean isoflavones are naturally occurring nonsteroidal phenolic plant compounds (9). Numerous studies confirmed their biological functions in promoting the growth of livestock, improving intestinal morphology, and enhancing antioxidant activity and immunity (10–13). Tannin is widely distributed in plant tissues and belongs to the secondary metabolite type and is considered to be an antinutritional factor. Tannin plays an important role in anti-inflammatory (14, 15), antioxidant (16), and antibacterial properties (17) and is reported to promote the growth and improve the health of broilers (18, 19). Therefore, tannins or soybean isoflavones have the potential to serve as an alternative to antibiotics in chicken production (20–22).

Tannin and soybean isoflavones have many similar biological functions, as both of them can promote the growth of poultry. However, there is less information on the application of tannins or soybean isoflavones and the comparison of the methods of application between these two plant extracts in Wenchang chickens. Therefore, this study aimed to evaluate the effects of dietary supplementation with dandelion tannins or soybean isoflavones on the growth performance, biochemical blood indexes, antioxidant function, and intestinal morphology and the immunity of Wenchang chickens.

## 2. Materials and methods

### 2.1. Animal ethics

All the experimental procedures applied in this study were reviewed and approved by the Experimental Animal Ethics Committee of Animal Husbandry and Veterinary Research Institute, Hainan Academy of Agricultural Sciences.

### 2.2. Experimental design and diets

The feeding experiments were conducted at the Institute of Animal Husbandry and Veterinary Medicine, Hainan Academy of Agricultural Sciences. Dandelion tannins (95% purity) and soybean isoflavones (26% purity) were purchased from Zhaoqing Baishike Biotechnology Co., Ltd. A total of 300 1-day-old female Wenchang chickens (average initial weight = 28.17 g) were randomly assigned into five groups, with six replicates per group and 10 broilers per replicate each. The chickens in the control group (Con) were fed a basal diet; the four experimental groups were fed a basal diet with different supplements: 300 mg/kg of dandelion tannin (DT1), 500 mg/kg of dandelion tannin (DT2), 300 mg/kg of soybean isoflavone (SI1), or 500 mg/kg of soybean isoflavone (SI2). During the experiment, the temperature inside the house was kept at 32°C and the humidity was set at 55%−65%. The experiment lasted 40 days. All experimental chickens were raised in three-layer cages, with two different types of lighting systems (artificial light and natural light) and access to food and water *ad libitum*. Body weight (BW) and feed intake were recorded at days 1 and 41 of the experiment. The average daily gain (ADG), average daily feed intake (ADFI), and feed weight ratio (F:G) were calculated based on the final weight and feed intake. The basic diet (Table 1) met the nutrition requirements of yellow feather broilers (NY/T 3645-2020).

**Table 1:** Feed and nutrient composition of the experimental chicken diet (as-fed basis, %).

**Feed ingredient**	**Content**	**Nutrients^a^**	**Content**
Corn	56.20	Crude protein (%)	20.00
Soybean meal	31.80	Metabolizable energy (MJ/kg)	12.18
Wheat	4.00	Methionine	0.32
Fish meal	1.00	Lysine	1.05
Soy oil	3.00	Calcium	0.85
Premix^b^	4.00	Non-phytate	0.40
Total	100		

a Nutrients are analyzed value.

b Premix provided the following per kilogram diet: 15,000 IU (international units) of vitamin A, 3,300 IU of vitamin D_3_, 20 mg of vitamin E, 6 mg of vitamin K_3_, 3 mg of vitamin B_1_, 8 mg of vitamin B_2_, 6 mg of vitamin B_6_, 0.03 mg of vitamin B_12_, 60 mg of niacin, 18 mg of calcium pantothenate, 1.5 mg of folic acid, 0.36 mg of biotin, 600 mg of choline chloride, 80 mg of Fe, 12 mg of Cu, 75 mg of Zn, 100 mg of Mn, 0.35 mg of I, and 0.15 mg of Se. The nutrition level indicators are calculated values.

### 2.3. Sample collection

At the end of the experiment, two chickens close to the average weight were selected for sample collection in each replicate. After weighing both the chickens, the blood sample was collected from the Pterygoid venous plexus using a coagulation tube and then centrifuged at 3,000 r/min (revolutions per minute) for 10 min to recover the serum.

After the blood was collected, the chickens were euthanized for duodenum, jejunum, and ileum sample collection. Each intestine was divided into two parts, one part of the intestinal segment (about 2 cm) was fixed in 10% paraformaldehyde fixative and the other part of the intestinal segment (about 2 g) was collected into 1.5 ml freezing tubes and stored at −80°C for RNA extraction. Cecal contents of samples were collected into 1.5 ml freezing tubes and stored at −80°C until analysis.

### 2.4. Serum biochemical indexes and antioxidant indexes

The concentrations of total protein, albumin, globulin, aspartate aminotransferase, uric acid, total cholesterol, triglycerides, and glucose in serum were determined using an automatic biochemical analyzer (Hitachi LABOSPECT 008 AS). The levels of malondialdehyde (MDA), superoxide dismutase (SOD), total antioxidant capacity (T-AOC), and catalase (CAT) in serum were determined using the commercial kits (A003-1-2, A001-3-2, A015-2-1, and A007-1-1; Nanjing Jiancheng Bioengineering Institute, Nanjing, China) according to the manufacturer's instructions.

### 2.5. Intestinal pH value

The intestinal pH value was determined using a pH meter (OHAUS, Beijing, China) by inserting the electrode into the anterior, middle, and posterior parts of the jejunum and the ileum. The average value of these three points was calculated.

### 2.6. Intestinal morphology

The fixed jejunum and ileum tissues were cut into sections with a thickness of 3–4 μm and stained with hematoxylin–eosin. Five photographs were selected randomly for each slide using a bright-field microscope (Eclipse Ci-L) at 40× magnification. The villus height (VH), the crypt depth (CD), and mucosal muscularis thickness (MMT) were analyzed using Image-Pro Plus 6.0. The villus height:crypt depth ratio (VH/CD) was calculated based on the VH and CD.

### 2.7. Quantitative real-time PCR

The total RNA was extracted from duodenum and ileum tissues using an RNA Extraction Kit (Tiangen, China) following the manufacturer's instructions. The complementary DNA (cDNA) was synthesized from 2 μg of total RNA by reverse transcription in a 20 μl reaction mixture using a reverse transcriptase kit (Tiangen, China). The reverse transcription procedure was employed as follows: 42°C for 15 min and 95°C for 3 min. The cDNA samples were stored at −20°C. The quantitative reverse transcription-polymerase chain reaction (qRT-PCR) was carried out on a Bio-Rad CFX96 Touch Real-Time PCR Detection System using the SYBR Green Real-time PCR Master Mix. Target gene expression was quantified using the 2^−ΔΔCt^ method. The real-time PCR procedure was employed as follows: denaturation at 95°C for 2 min, followed by 39 cycles at 95°C for 0.5 s and 60°C for 10 s. The primers are listed in Table 2.

**Table 2:** Primers used for quantitative real-time polymerase chain reaction (PCR) in this study.

**Gene**	**Primer sequence (5^′^-3^′^)**
β-ACTIN	F: ACCTGAGCGCAAGTACTCTGTCT R: CATCGTACTCCTGCTTGCTGAT
TNF-α	F: AGTGCTGTTCTATGACCGCC R: CGCTCCTGACTCATAGCAGA
TGF-β4	F: AGGATCTGCAGTGGAGTGGAT R: CCCCGGGTTGTGTTGGT
ZO-1	F: GCCAGCCATCATTCTGACTCCAC R: GTACTGAAGGAGCAGGAGGAGGAG
Claudin-1	F: GATCCAGTGCAAGGTGTACGA R: AAAGACAGCCATCCGCATCT
Occludin	F: ACGGCAGCACCTACCTCAA R: GGGCGAAGAAGCAGATGAG

TNF-α, tumor necrosis factor alpha; TGF-β, transforming growth factor beta; ZO, tight junction protein; F, forward primer; R, reverse primer.

### 2.8. Cecal microbiota

The cecal content was collected into a sterile Eppendorf (EP) tube, immediately frozen in liquid nitrogen, and stored at −80°C until analysis. The DNA of cecal content was extracted by cetyltrimethylammonium bromide (CTAB) method, and the purity and concentration of DNA were detected by agarose gel electrophoresis. The V3-V4 variable region was amplified using the primer (Forward: CCTAYGGGRBGCASCAG; Reverse: GGACTACNNGGGTATCTAAT). The PCR system (30 μl) was comprised of 15 μl of Phusion High-Fidelity PCR Master Mix (2×), 1 μl of each primer (1 μM), 10 μl of genomic DNA (gDNA) (1 ng/μl), and 3 μl of water. The PCR procedure was employed as follows: 98°C predenaturation for 1 min, followed by 30 cycles of 98°C for 10 s, 50°C for 30 s, 72°C for 30 s, and finally 72°C for 5 min. The PCR products were diluted to the same concentration. Then, 2% agarose gel electrophoresis was used to purify the PCR products. A library building kit (Illumina, San Diego, USA) was used for library construction. After the library was qualified, a NovaSeq 6000 was employed for online sequencing (Entrust Nuohe Zhiyuan Biological Information Technology Co., Ltd).

### 2.9. Statistical analysis

All data were analyzed using SPSS version 26.0 (IBM Corp., USA). Data were expressed as the means ± standard deviation. Between-group differences were analyzed using one-way analysis of variance (ANOVA), with a *P*-value of <0.05 as the significance level, and Duncan's multiple range test was used for multiple comparisons.

## 3. Results

### 3.1. Growth performance

The final body weight and average daily gain were higher in the DT2 and SI1 groups (*P* < 0.05) than in the Con group. Additionally, there were no differences in the ADFI or feed weight ratio in the experimental groups as in the Con group (*P* > 0.05). However, the feed weight ratio in the experimental groups exhibited a downward trend (0.05 < *P* < 0.1) compared to the controls (Table 3).

**Table 3:** Effects of dietary supplementation with dandelion tannins or soybean isoflavones on the growth performance of Wenchang chickens.

**Items**	**Control**	**DT1**	**DT2**	**SI1**	**SI2**	***P*-value**
Initial BW, g	28.17 ± 0.26	28.17 ± 0.26	28.17 ± 0.26	28.17 ± 0.26	28.17 ± 0.26	1.00
Final BW, g	495.22 ± 35.24^b^	531.19 ± 35.94^ab^	548.24 ± 23.19^a^	548.30 ± 22.76^a^	525.81 ± 32.34^ab^	0.04
ADG, g/d	11.68 ± 0.88^b^	12.58 ± 0.90^ab^	13.00 ± 0.58^a^	13.00 ± 0.57^a^	12.44 ± 0.80^ab^	0.03
ADFI, g/d	25.59 ± 1.46	26.41 ± 1.98	27.73 ± 1.21	26.99 ± 1.41	25.71 ± 1.79	0.14
F/G, g/g	2.21 ± 0.30	2.10 ± 0.09	2.13 ± 0.03	2.08 ± 0.07	2.07 ± 0.13	0.79
Mortality rate, %	20.00	6.67	6.67	10.00	10.00	0.33

BW, body weight; ADG, average daily gain; ADFI, average daily feed intake; F/G, feed intake/body weight gain. DT1, basal diet +300 mg/kg dandelion tannins; DT2, basal diet +500 mg/kg dandelion tannins; SI1, basal diet +300 mg/kg soybean isoflavones; SI2, basal diet +500 mg/kg soybean isoflavones.

The data were expressed as the means ± standard deviation.

ab Marks indicate statistically significant differences (*P* < 0.05).

### 3.2. Serum biochemical indexes

**Figure 1:**
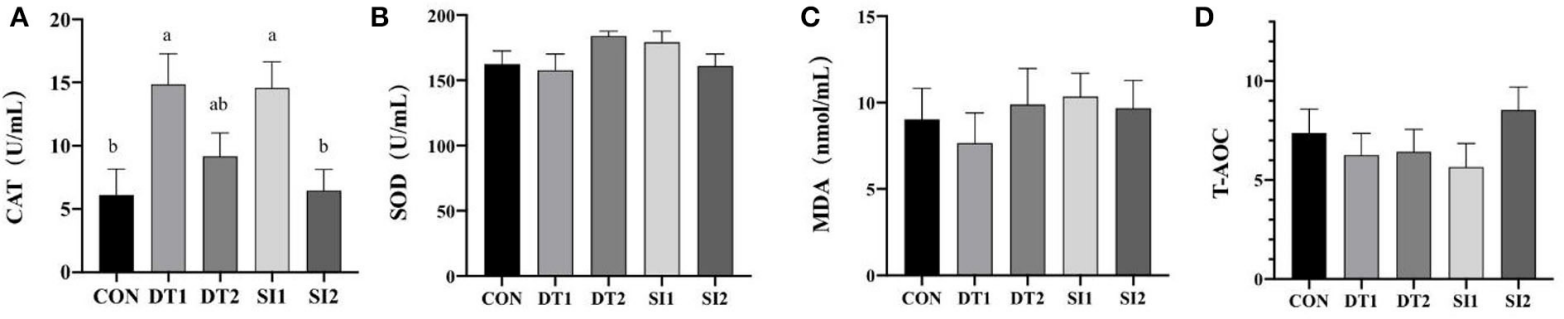
**(A–D)** Effects of dietary dandelion tannins or soybean isoflavones on serum antioxidant indexes. ^*a,b,c*^Marks indicate statistically significant differences (*P* < 0.05).

As shown in Table 4, there was no difference in the level of serum total protein, globulin, uric acid, or total cholesterol among the groups (*P* > 0.05). However, the level of serum albumin was increased in the DT2, SI1, and SI2 groups compared to the Con group (*P* < 0.05). In addition, the SI1 group was higher than the Con group in the levels of serum aspartate aminotransferase and glucose (*P* < 0.05). Similarly, the level of triglycerides was higher in the DT1 group (*P* < 0.05) than in the Con group. As shown in Figure 1, the serum CAT level in the DT1 and SI1 groups was higher than that in the Con group (*P* < 0.05). There was no significant difference in the serum MDA, SOD, or T-AOC levels among the groups (*P* > 0.05).

**Table 4:** Effects of dietary supplementation with dandelion tannins or soybean isoflavones on serum biochemical indexes of Wenchang chickens.

**Items**	**Control**	**DT1**	**DT2**	**SI1**	**SI2**	***P*-value**
Total protein, g/L	42.24 ± 6.85	40.30 ± 6.19	40.85 ± 3.83	39.72 ± 4.64	40.78 ± 4.96	0.82
Albumin, g/L	12.72 ± 1.28^b^	13.67 ± 1.55^ab^	14.05 ± 1.00^a^	14.07 ± 1.00^a^	14.13 ± 0.84^a^	0.04
Globulin, g/L	29.52 ± 6.56	26.63 ± 6.26	26.79 ± 3.28	25.65 ± 3.99	26.65 ± 4.73	0.66
Aspartate aminotransferase, U/L	229.30 ± 23.99^b^	251.75 ± 26.36^b^	245.55 ± 28.85^b^	289.09 ± 32.59^a^	254.08 ± 30.75^b^	<0.01
Uric acid, μmol/L	210.30 ± 96.15	232.67 ± 67.15	205.09 ± 54.40	192.45 ± 49.46	165.08 ± 36.69	0.13
Total cholesterol, mmol/L	3.04 ± 0.66	2.79 ± 0.52	2.93 ± 0.46	3.02 ± 0.34	2.89 ± 0.50	0.77
Triglycerides, mmol/L	0.65 ± 0.13^ab^	0.77 ± 0.21^a^	0.65 ± 0.16^ab^	0.54 ± 0.13^b^	0.54 ± 0.17^b^	0.03
Blood glucose, mmol/L	6.86 ± 3.48^b^	6.93 ± 3.13^b^	8.92 ± 2.07^ab^	10.18 ± 1.70^a^	9.47 ± 1.25^ab^	0.02

DT1, basal diet +300 mg/kg dandelion tannins; DT2, basal diet +500 mg/kg dandelion tannins; SI1, basal diet +300 mg/kg soybean isoflavones; SI2, basal diet +500 mg/kg soybean isoflavones.

The data were expressed as the means ± standard deviation.

a, b Marks indicate statistically significant differences (*P* < 0.05).

### 3.3. Intestinal pH and histomorphology

As shown in Table 5, the ileum pH value was the lowest (*P* < 0.05) in the DT2 and SI1 groups. As shown in Table 6, an increasing trend (*P* < 0.05) in jejunum VH and MMT was found in the DT2 and SI1 groups compared to the Con group. The jejunum CD was decreased (*P* < 0.05) in the DT1 and DT2 groups compared to the Con group. In addition, the jejunum V:C was increased significantly in the DT2 group compared to the Con group (*P* < 0.05). However, no significant difference was observed in the jejunum V:C between the SI1 group and the SI2 group (*P* > 0.05). Similarly, no significant differences were observed in the ileum VH, CD, MMT, and V:C among the groups (*P* > 0.05).

**Table 5:** Effects of dietary supplementation with dandelion tannins or soybean isoflavones on the intestinal pH of Wenchang chickens.

**Item**	**Control**	**DT1**	**DT2**	**SI1**	**SI2**	***P*-value**
Ileum pH	6.76 ± 0.11^ab^	6.87 ± 0.29^a^	6.62 ± 0.12^b^	6.61 ± 0.26^b^	6.82 ± 0.33^ab^	0.04
Jejunum pH	6.44 ± 0.10	6.49 ± 0.25	6.36 ± 0.17	6.31 ± 0.36	6.44 ± 0.11	0.32

DT1, basal diet +300 mg/kg dandelion tannins; DT2, basal diet +500 mg/kg dandelion tannins; SI1, basal diet +300 mg/kg soybean isoflavones; SI2, basal diet +500 mg/kg soybean isoflavones.

The data were expressed as the means ± standard deviation.

a, b Marks indicate statistically significant differences (*P* < 0.05).

**Table 6:** Effects of dietary supplementation with dandelion tannins or soybean isoflavones on jejunum and ileum histomorphology of Wenchang chickens.

**Item**	**Control**	**DT1**	**DT2**	**SI1**	**SI2**	***P-*value**
**Jejunum**						
VH, mm	1.23 ± 0.26^a^	1.03 ± 0.20^b^	1.31 ± 0.22^a^	1.32 ± 0.18^a^	1.18 ± 0.25^ab^	0.02
CD, mm	0.22 ± 0.04^a^	0.16 ± 0.03^b^	0.18 ± 0.04^b^	0.21 ± 0.02^a^	0.19 ± 0.03^ab^	<0.01
MMT, mm	1.48 ± 0.28^a^	1.26 ± 0.24^b^	1.54 ± 0.21^a^	1.58 ± 0.13^a^	1.41 ± 0.27^ab^	0.01
V:C	5.77 ± 1.25^b^	6.41 ± 1.52^ab^	7.51 ± 1.38^a^	6.22 ± 0.91^b^	6.38 ± 1.55^ab^	0.05
**Ileum**						
VH, mm	0.91 ± 0.15	0.84 ± 0.16	0.93 ± 0.18	0.97 ± 0.14	0.95 ± 0.14	0.19
CD, mm	0.21 ± 0.03	0.18 ± 0.04	0.19 ± 0.03	0.21 ± 0.02	0.21 ± 0.03	0.07
MMT, mm	1.20 ± 0.20	1.07 ± 0.18	1.17 ± 0.18	1.24 ± 0.16	1.24 ± 0.16	0.06
V:C	4.29 ± 0.68	4.79 ± 1.53	4.93 ± 1.37	4.62 ± 0.48	4.50 ± 0.55	0.64

DT1, basal diet +300 mg/kg dandelion tannins; DT2, basal diet +500 mg/kg dandelion tannins; SI1, basal diet +300 mg/kg soybean isoflavones; SI2, basal diet +500 mg/kg soybean isoflavones; VH, villus height; CD, crypt depth; MMT, mucosal muscularis thickness; V:C, villus height:crypt depth ratio.

The data were expressed as the means ± standard deviation.

a, b Marks indicate statistically significant differences (*P* < 0.05).

### 3.4. The mRNA expression level of genes

**Figure 2:**
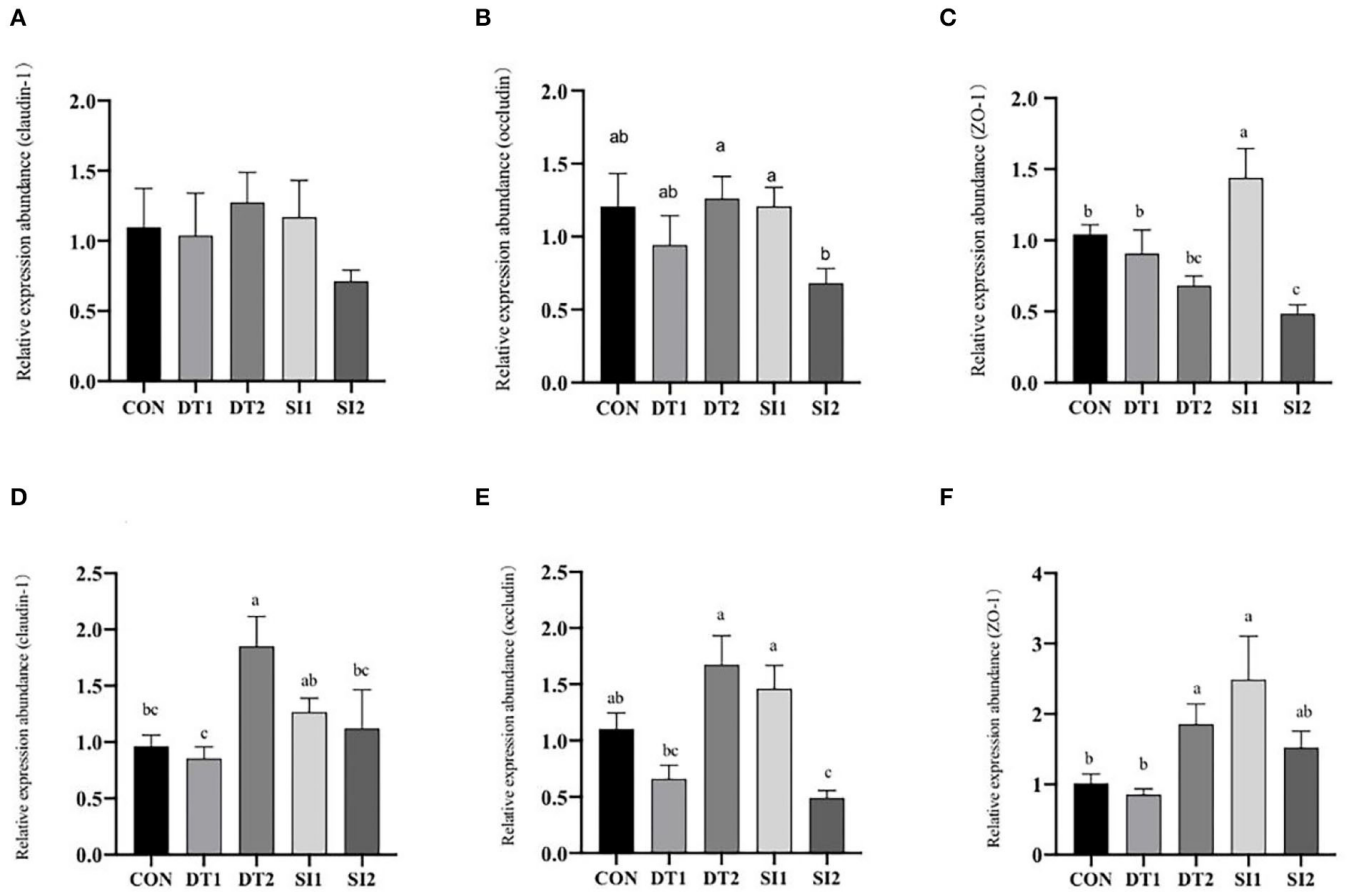
Effects of dietary dandelion tannins or soybean isoflavones on the messenger RNA (mRNA) expression of tight junction-related genes in duodenum **(A–C)** and ileum **(D–F)**. ^*a,b,c*^Marks indicate statistically significant differences (*P* < 0.05).

As shown in Figure 2, the mRNA expression level of *occludin* in the duodenum was the highest in the DT2 groups. The mRNA level of *ZO-1* in the SI1 group was significantly upregulated (*P* < 0.05) compared to the Con group. In the ileum, the mRNA expression level of *occludin* was higher in the DT2 and SI1 groups than in the Con group. The mRNA expression level of *ZO-1* was significantly higher in the DT2 and SI1 groups than in the Con group (*P* < 0.01). Relative to the Con group, the mRNA expression level of *claudin-1* was significantly increased in the DT2 group (*P* < 0.01).

**Figure 3:**
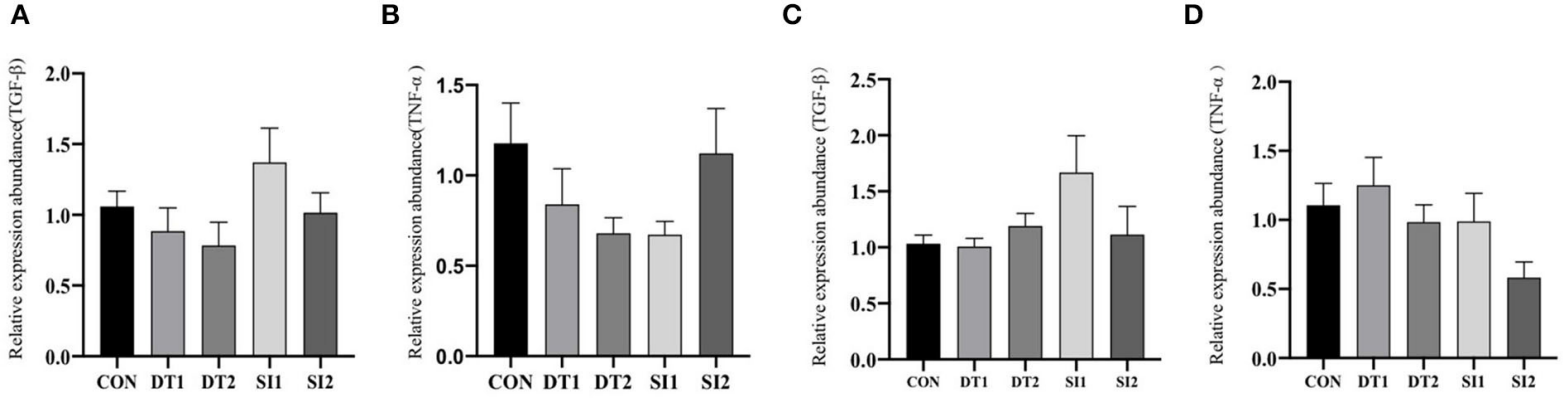
Effects of dietary dandelion or soybean isoflavones on the messenger RNA (mRNA) expression of inflammation-related genes in duodenum **(A, B)** and ileum **(C, D)**.

Dietary supplementation with dandelion tannins or soybean isoflavones has no significant effect on the mRNA levels of tumor necrosis factor-alpha (*TNF-*α) and transforming growth factor beta (*TGF-*β) in the duodenum and ileum (Figure 3).

### 3.5. Cecal microbiota

**Figure 4:**
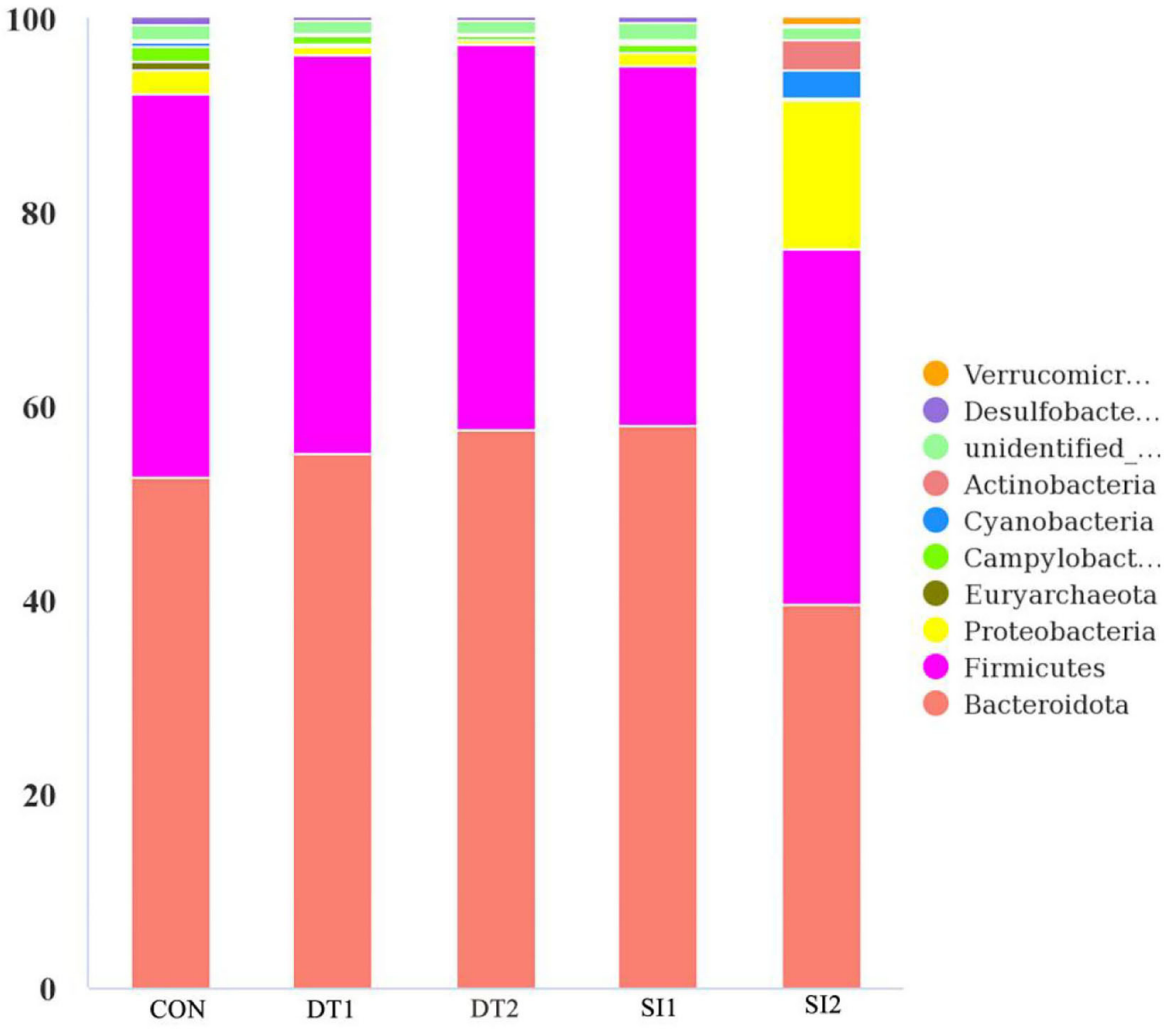
The top 10 most abundant taxa of cecal microbiota at the phylum level.

**Figure 5:**
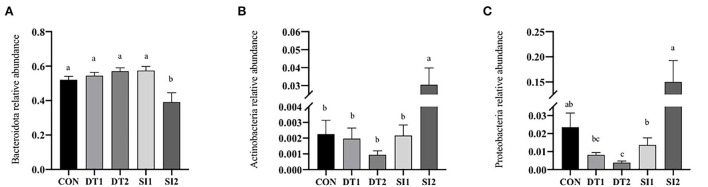
Effects of dietary dandelion tannins and soybean isoflavones of cecal microbiota at the phylum level. The relative abundance of Bacteroidota **(A)**, Actinobacteria **(B)**, and Proteobacteria **(C)**. ^*a,b,c*^Marks indicate statistically significant differences (*P* < 0.05).

The Chao1 and abundance-based coverage estimator (ACE) indexes were higher in the SI2 group than in the Con group. By contrast, the Shannon and Simpson indexes were significantly decreased in the DT2 and SI1 groups (*P* < 0.01; Table 7). At the phylum level, the top 10 most abundant taxa of cecal microbiota were Bacteroidota, Firmicutes, Proteobacteria, Euryarchaeota, Campylobacterota, Cyanobacteria, Actinobacteria, unidentified-bacteria, Desulfobacterota, and Verrucomicrobiota. The abundance of Bacteroidota and Firmicutes accounted for more than 90% of the total bacteria found in the cecal microbiota (Figure 4). The relative abundance of Actinobacteria was the highest, while the relative abundance of Bacteroidota was decreased significantly in the SI2 group compared to the Con group (*P* < 0.01). The relative abundance of Proteobacteria was decreased in the DT2 group compared to the Con group (*P* < 0.01; Figure 5).

**Table 7:** The Effects of dietary supplementation with dandelion tannins or soybean isoflavones on the α-diversity indexes of cecal microbiota.

**Item**	**Control**	**DT1**	**DT2**	**SI1**	**SI2**	***P*-value**
Shannon	5.25 ± 0.37^a^	5.22 ± 0.34^a^	4.68 ± 0.34^b^	4.76 ± 0.40^b^	5.31 ± 0.43^a^	< 0.01
Simpson	0.91 ± 0.03^a^	0.91 ± 0.03^ab^	0.87 ± 0.03^c^	0.88 ± 0.02^bc^	0.90 ± 0.03^ab^	<0.01
Chao1	854.34 ± 115.52^b^	863.12 ± 132.77^b^	781.54 ± 92.46^b^	812.25 ± 186.09^b^	977.90 ± 125.53^a^	<0.01
ACE	718.36 ± 86.56^b^	710.86 ± 97.51^b^	644.77 ± 64.83^b^	657.22 ± 146.01^b^	816.91 ± 99.40^a^	<0.01

DT1, basal diet +300 mg/kg dandelion tannins; DT2, basal diet +500 mg/kg dandelion tannins; SI1, basal diet +300 mg/kg soybean isoflavones; SI2, basal diet +500 mg/kg soybean isoflavones.

a, b Marks indicate statistically significant differences (*P* < 0.05).

**Figure 6:**
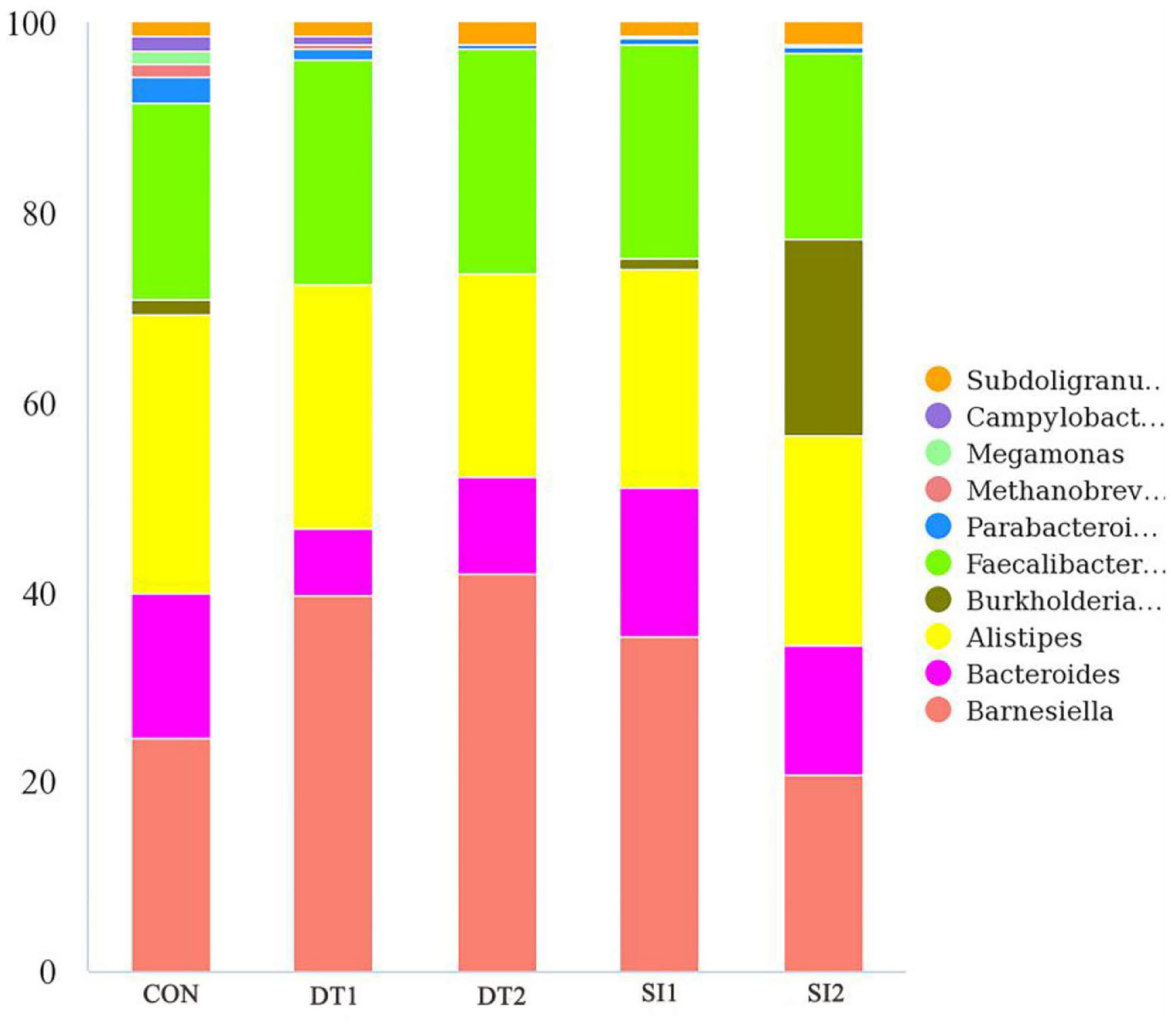
The top 10 most abundant taxa of cecal microbiota at the genus level.

**Figure 7:**
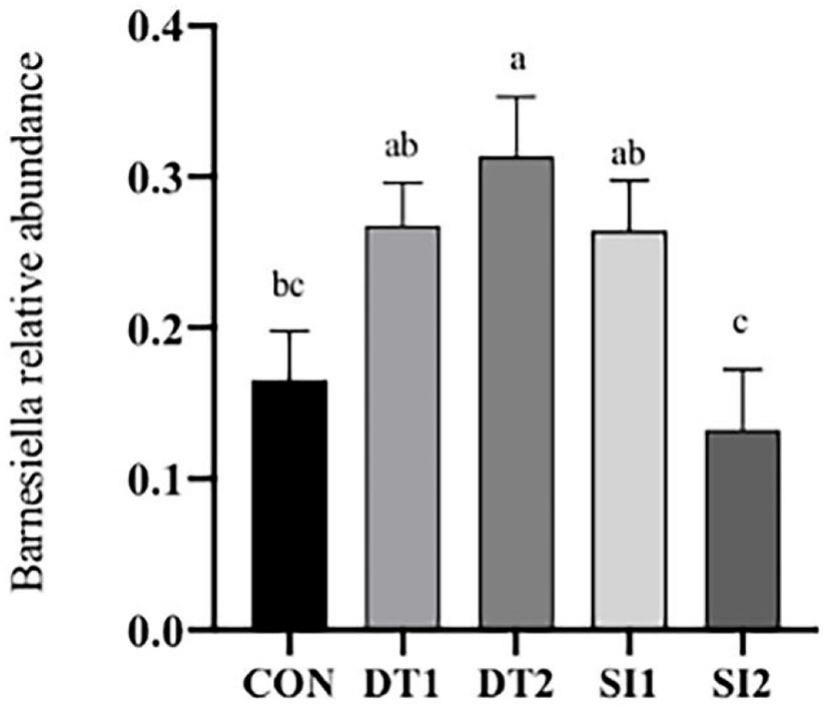
Effects of dietary dandelion tannins or soybean isoflavones on cecal microbiota at the genus level. ^*a,b,c*^Marks indicate statistically significant differences (*P* < 0.05).

**Figure 8:**
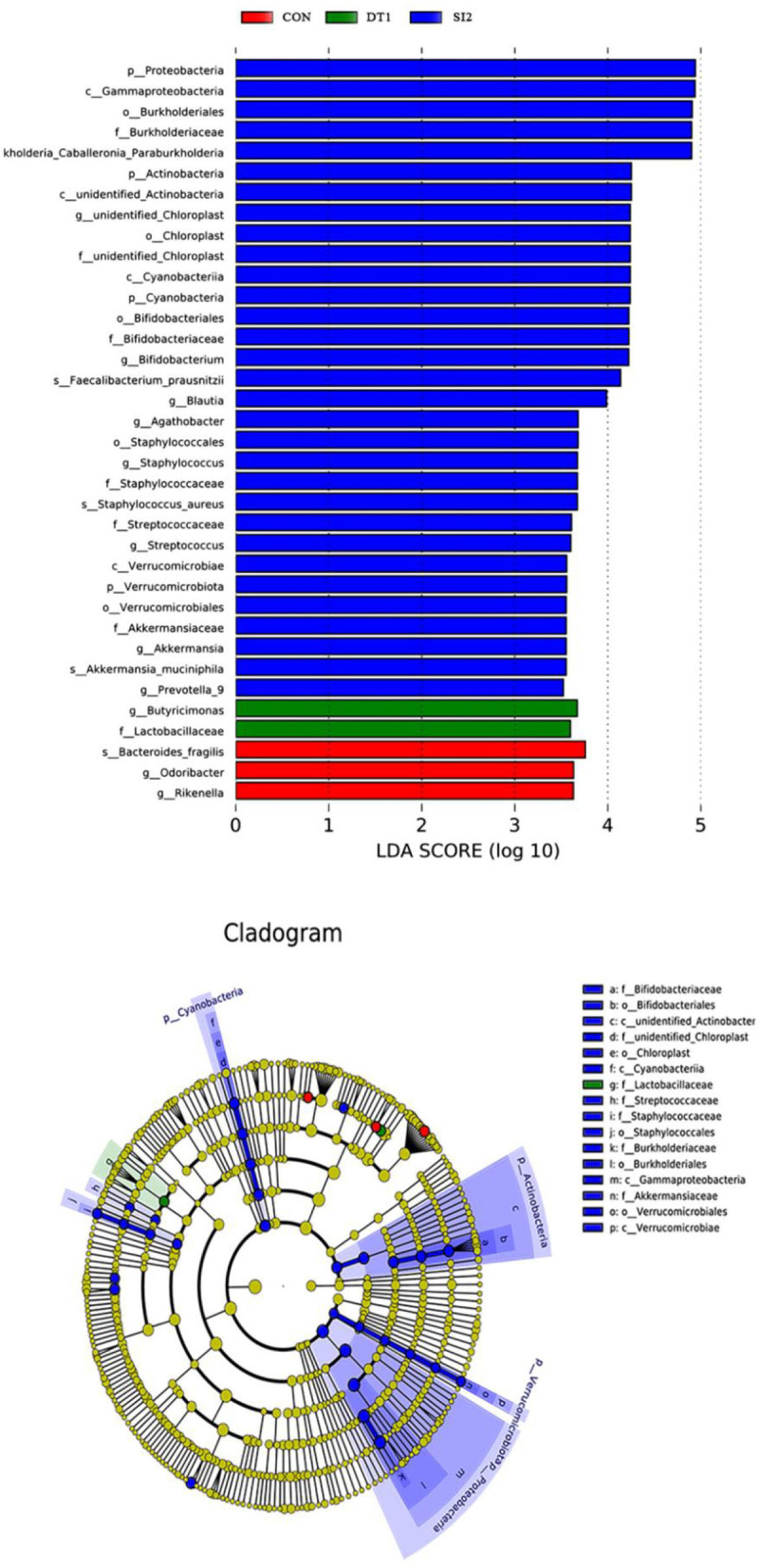
Linear effect size (LDA) effect size (LEfSe) analysis at the family level with the threshold set to 3.5.

At the genus level (Figure 6), the most dominant microbiota (>5%) were *Barnesiella* (22.88%), *Bacteroides* (8.58%), *Alistipes* (16.87%), and *Faecalibacterium* (15.39%). The relative abundance of *Barnesiella* was significantly increased in the DT2 group compared to the Con group (Figure 7). The linear discriminant analysis (LDA) effect size (LEfSe) analysis indicated that DT1 increased the relative abundance of *Lactobacillaceae*, while SI2 increased the relative abundance of *Bifidobacteriaceae, Unidentified-chloroplast, Streptococcaceae, Staphylococcaceae, Burkholderiaceae, and Akkermansiaceae* (Figure 8).

## 4. Discussion

The use of antibiotics as growth promoters in chicken production proved to be harmful to human health (23). Therefore, alternative innovative methods are urgently needed to be implemented to increase chicken production. The beneficial effects of tannins or soybean isoflavones have been well recognized for improving growth performance (18, 21, 24). In the present study, 1-day-old chickens were selected as models to evaluate the effects of dietary supplementation with dandelion tannins or soybean isoflavones on Wenchang chickens. Consistent with published studies (25, 26), an increase in BW and ADG was observed in the DT2 and SI1 groups. These results indicate that the growth performance improved with dandelion tannins' or soybean isoflavones' supplementation. The optimal growth performance is directly linked to the health of the intestine (27). Evidence showed that dandelion tannins could improve intestinal morphology and regulate the composition of intestinal microbiota (28, 29). These results indicate that dandelion tannins could maintain the health status of the intestine, thereby indirectly promoting the growth of the broiler (30). Evidence showed that soybean isoflavones could improve growth performance by regulating the hormone level related to growth and improving the metabolism and utilization of substances (9, 31). However, in the present study, a diet supplemented with 300 mg/kg of dandelion tannin or 500 mg/kg of soybean isoflavone did not lead to a significant improvement in growth performance, indicating that the effects of tannins or flavonoids on growth performance are dosage-dependent.

Blood biochemical indicators reflect the health status of the animal. Albumin is mainly produced by the liver, which indicates an improvement in protein synthesis at a higher level, whereas it causes liver damage at a lower level (32, 33). Blood glucose reflects energy metabolism (34) and is the main source of energy (35). Maintaining a normal blood glucose level is important for the physiological function of animals (36). In the present study, the serum albumin and blood glucose levels in experimental groups were increased, which was consistent with the changes in growth performance. Triglycerides are synthesized in the liver and adipose tissues. Most of the tissues in the human body can use the energy provided by the fatty acids and glycerol released by triglyceride decomposition (37). However, studies reported that the intake of meat with a high level of triglycerides is not beneficial to human health (38). In the present study, soybean isoflavones could decrease the serum triglyceride levels. Consequently, the results suggested that an appropriate level of dandelion tannins or soybean isoflavones in diets can promote growth by improving protein synthesis and lipid metabolism.

Poultry growth can be hampered by oxidative stress induced by nutrition, environment, disease, and other factors (39). Antioxidation is one of the most important biological functions of dandelion tannins or soybean isoflavones (40, 41). The antioxidant enzymes, including SOD, CAT, and glutathione peroxidase (GSH-Px), can effectively clean up free radicals and restore the balance between oxidants and antioxidants. As the product of lipid peroxidation, MDA indirectly reflects the extent of oxidative damage. Previous studies showed that the addition of dandelion tannins or plant flavonoids to a broiler diet could improve the serum antioxidant capacity (42, 43). Our study also found that the serum CAT levels became significantly higher with dandelion tannins' or soybean flavonoids' supplementation, suggesting that the dietary supplementation with low-dose dandelion tannins or soybean isoflavones could improve the serum antioxidant status of broilers.

The intestinal pH value is an important index reflecting the digestive environment *in vivo*. When the pH is close to 7 or slightly higher, it is conducive to the growth of pathogens (44). In contrast, a lower intestinal pH promotes the growth of beneficial microorganisms and nutrient absorption (45, 46). In the present study, the pH of the ileum in the DT2 and SI1 groups was significantly decreased. These results were in agreement with those of the previous study, where it was reported that adding a plant-based supplement (Galibiotic™) to broiler feed significantly decreased intestinal pH (44). Similar results were also found with the addition of turmeric meal individually or in combination with a wheat-soybean meal (47). Furthermore, 500 mg/kg of dandelion tannins and 300 mg/kg of soybean isoflavones had brought about the effect of reducing the intestinal pH to a certain degree, potentially improving nutrient absorption and promoting the growth of chickens.

The intestinal histomorphology can reflect the health status of the intestine, as well as the digestion and absorption of nutrients (48). To understand the underlying mechanism of the growth-promoting effect of dandelion tannins and soybean isoflavones, the intestinal villus length and crypt depth were measured (49). In this study, the jejunal villus height to crypt depth ratio significantly increased in the DT2 group than in the Con group, and the jejunal villus length was higher in the DT2 and SI1 groups, which indicates that dandelion tannins or soybean isoflavones could enhance the function of the small intestine (50). In line with our study, evidence showed that the addition of tannins to the diet significantly increased the jejunal villus length and alleviated the negative effects of heat stress on intestinal histomorphology (42). In addition, soybean isoflavones were shown to increase the villus length and protect intestinal health (13). The intestine is not only an absorptive organ but also an important immune organ (51). The mucosal muscularis is the first barrier to preventing the destruction of intestines by pathogenic microorganisms (52). *Occludin* and *claudin*, two transmembrane proteins, are crucial components of tight junctions (53, 54) and play an important role in the maintenance of the epithelial barrier (55). In the present study, the jejunal muscularis thickness was higher in the DT2 and SI1 groups. The mRNA expression of *ZO-1* in the duodenum was higher in the SI1 group and those of *occludin, ZO-1*, and *claudin-1* were higher in the DT2 and SI1 groups. Collectively, these results indicate that dandelion tannins or soybean isoflavones contribute to the growth of chickens which might be attributed to the improved intestinal health.

Microbiota is a highly complex microbial community that directly affects the health, immunity, and productivity of animals (56). Previous studies found that intestinal microbes could affect the feed conversion ratio of chickens, whereas chickens with greater feed conversion ratios had lower α-diversity of gut microbiota (57, 58). In this study, there was lower community richness and diversity in cecum in the DT2 and SI1 groups. This may be one of the reasons for the improved growth performance of Wenchang chickens. It was reported that Firmicutes and Bacteroidetes are the major phyla of cecal microbiota, comprising of up to 90% of the total microbiota (59, 60). The Firmicutes primarily synthesizes butyrate and propionate, which are the major sources of energy for intestinal cells (61). Evidence showed that the abundance of Firmicutes was positively correlated with weight gain (62). There is a mutualistic relationship between Bacteroidetes and the animal host. Bacteroidetes helps the body to gain energy from the degradation of carbohydrates (63, 64) and maintain a healthy gut (65, 66). In this study, Bacteroidetes and Firmicutes accounted for more than 90% of intestinal bacteria in 40-day-old Wenchang chickens, which was in agreement with the literature (67). In addition, there were seven species of bacteria with a significant difference at the family level, according to the LDA effect size analysis. *Lactobacillaceae* can protect the gut by inhibiting pathogen colonization (68, 69) and have a positive effect on the feed efficiency of chickens (70). *Bifidobacteriaceae* have shown beneficial effects in an animal, such as promoting nutrient absorption and improving immunity (71). *Akkermansiaceae* belong to the phylum *Verrucomicrobia* and are considered anti-inflammatory bacteria (72). The abundance of *Akkermansiaceae* is negatively correlated with obesity, which in turn promotes the occurrence of inflammatory diseases (73). As a corollary, increasing the abundance of *Akkermansiaceae* could reduce inflammation (74). *Staphylococcaceae* belong to the Firmicutes and most of them are nonpathogenic, but a few species can cause disease. These bacteria are widespread in farmed chickens and are capable of undergoing fermentative metabolism (75, 76). This study demonstrates that dietary supplementation with dandelion tannins or soybean isoflavones regulated the gut microbiota composition related to the growth performance of Wenchang chickens.

## 5. Conclusions

This study demonstrates that dietary supplementation with dandelion tannins or soybean isoflavones improved the growth performance, serum biochemical indexes, serum antioxidant capacity, and intestinal health of 40-day-old Wenchang chickens. Based on the results, this study recommends that the dosage of dandelion tannins and soybean isoflavones added to diets of 40-day-old Wenchang chickens be 500 and 300 mg/kg, respectively. Our findings provide new insights into the potential of using natural plant extracts as feed additives.

## Data availability statement

The datasets presented in this study can be found in online repositories. The names of the repository/repositories and accession number(s) can be found at: http://www.ncbi.nlm.nih.gov/bioproject/893339.

## Ethics statement

The animal study was reviewed and approved by the Experimental Animal Ethics Committee of Animal Husbandry and Veterinary Research Institute, Hainan Academy of Agricultural Sciences.

## Author contributions

XL prepared the manuscript and collected some data. RS, YG, YO, QQ, YX, XW, CH, and SJ collected the samples. GZ and LW were responsible for the design and direction of the experiment. All authors have read and agreed to the published version of the manuscript.
